# Otoneurological assessment and quality of life of individuals with complaints of dizziness and temporomandibular disorders: a case-control study

**DOI:** 10.1016/j.bjorl.2022.04.005

**Published:** 2022-05-20

**Authors:** Mônica Claudino Medeiros Honorato, Luiz Felipe Tavares, Henrique de Paula Bedaque, Erika Barioni Mantello, Erika Oliveira de Almeida, Karyna Myrelly Oliveira Bezerra de Figueiredo Ribeiro, Lidiane Maria de Brito Macedo Ferreira

**Affiliations:** a Universidade Federal do Rio Grande do Norte, Departamento de otorrinolaringologia, Natal, RN, Brasil; b Universidade Federal do Rio Grande do Norte, Departamento de fisioterapia, Natal, RN, Brasil; c Universidade Federal do Rio Grande do Norte, Departamento de fonoaudiologia, Natal, RN, Brasil; d Universidade Federal do Rio Grande do Norte, Departamento de odontologia, Natal, RN, Brasil

**Keywords:** Temporomandibular joint, Dizziness, Quality of life

## Abstract

**Objectives:**

To evaluate the impact of temporomandibular disorders on the quality of life of patients with dizziness.

**Methods:**

An observational, case-control study evaluated 60 individuals with dizziness (20 cases and 40 controls), who were matched for gender and age. The individuals underwent to anamnesis, overall physical and otoneurological examination, tonal and vocal audiometry and impedanciometry, video head impulse test and the dizziness handicap inventory questionnaire.

**Results:**

The otoscopy was normal for all patients. There was an association between the presence of temporomandibular disorders and aural fullness (*p* < 0.01) and otalgia (*p* < 0.01). Audiometry was normal in 90% of the patients in the case group, with a significant association between temporomandibular disorders and normal audiometry (*p* < 0.01). The video head impulse test findings were normal in 66% of the patients in the case group and 45% of the control group, and there was no association between having temporomandibular disorders and vestibular alterations at the video head impulse test (*p* = 0.12). There were significant differences in total dizziness handicap inventory and in the functional and emotional domains (*p* < 0.01), with higher scores in the control group.

**Conclusion:**

Aural fullness and otalgia symptoms are associated with temporomandibular disorders in patients with dizziness, and there is an association between normal complementary audiological tests and temporomandibular disorders. Vestibular alterations are not associated with temporomandibular disorders. However, patients with dizziness and without temporomandibular disorders showed greater quality of life impairment.

**Level of evidence:**

3. Original case-control study.

## Highlights

• Aural fullness and otalgia symptoms are associated with temporomandibular disorders in patients with dizziness.
• Patients with dizziness and without temporomandibular disorders have a greater impact on quality of life.
• The most affected quality of life domains are the functional and emotional ones.

## Introduction

The temporomandibular joint (TMJ) is located between the temporal bone and the mandible.^1^ The adequate functioning of this joint is essential for the performance of functional movements such as speaking and chewing.^2^ Alterations in the mastication muscles, the TMJ, and/or associated structures can lead to temporomandibular disorders (TMD),^3^ which affect approximately 5%–12% of the population.^4^ The main symptoms of TMD are facial pain, headache, and pain in the TMJ and mastication muscles topography.^5^ Moreover, otoneurological symptoms such as tinnitus, dizziness, vertigo, otalgia, hearing loss and aural fullness may be present in individuals with TMD,^3^, ^6^ with a prevalence of dizziness of 22% and vertigo 40%.^7^ TMDs contribute to public health costs and have a direct impact on society.^8^

Regarding dizziness, according to the Barany Society,^9^ dizziness can be defined as a sensation of disturbance or impaired spatial orientation, without a false or distorted sense of movement, while vertigo is differentiated by being characterized as a false sensation of self-motion and a false sensation that the visual environment is rotating or flowing. However, the American Society of Otorhinolaryngology considers dizziness a universal term that includes vertigo as a subtype.^5^

Dizziness affects 15%–35% of the world’s population at some point during their lifetime.^10^ Individuals with dizziness or vertigo have reported requiring rest from work, interruption of daily activities and being afraid of going out home, in addition to having a lower quality of life.^10^ Associations between alterations in spinal movements, cervicogenic dizziness, and TMD have been recently reported in the literature.^11^ There is no consensus about a possible causal relationship between dizziness and TMD. Controversial theories try to explain the relationship between otoneurological symptoms and TMD,^12^ with the main ones indicating a possible transmission of mechanical energy from the TMJ to the middle ear through the discomalleolar ligament, irritation of the auriculotemporal nerve and hypertonicity of the muscles innervated by the trigeminal nerve. 13, 14

Symptoms such as dizziness may be present in individuals with TMD and even interfere with the performance of daily activities, generating emotional, physical and functional consequences.^15^ Studies that identify risks and associated factors are important to improve the management of patients with otoneurological symptoms and TMD. Therefore, this study aimed to evaluate the impact that TMD generates on the quality of life of patients with dizziness.

## Methods

### Study design and location

This is a case-control observational study, carried out in the Dentistry Department of a Federal University and in a tertiary hospital, from August 2019 to October 2020. This study was approved by the Research Ethics Committee. All participants signed the free and informed consent form (FICF).

### Participants

Individuals of both genders, aged 18–65 years, with a complaint of dizziness, defined according to the American Society of Otorhinolaryngology, were included in the study.^9^ Individuals with impaired cognitive ability measured by the Mini-Mental State Examination, with a score below 24 points;^16^ a history of head trauma related to the origin of the orofacial pain, confounding the diagnosis of TMD; intracranial disorders that required treatment for TMD within the last three months; other causes of orofacial pain such as caries, periodontal diseases, neuropathies and fibromyalgia, as well as patients who did not voluntarily agree to participate or who did not sign the free and informed consent form, were not included in the study.

Cases were defined as patients diagnosed with TMD (articular, muscular or mixed) by a dental surgeon with experience and skills to apply the Research Diagnostic Criteria for Temporomandibular Disorders (RDC/TMD) Axis I.^17^ The individuals were recruited from the Integrated Care Center for Patients with Stomatognathic System Dysfunction (CIADE, *Centro Integrado de Atendimento a Portadores de Disfunção do Aparelho Estomatognático*) extension project, located at a Federal University and referred after dental and physical therapy evaluation for otoneurological evaluation at the university hospital. The patient control group had their information obtained from the medical records of the neurotology outpatient clinic of the same hospital.

The sample size was defined based on the prevalence of 10% of TMD in the population,^18^ which represents 353,416 inhabitants of the state where the research was carried out, based on the population estimated in 2020 by the Brazilian Institute of Geography and Statistics (IBGE, *Instituto Brasileiro de Geografia e Estatística*). Therefore, considering a standard error of 4%, a confidence interval of 95% and a maximum expected percentage of people with dizziness due to TMD of 0.75%^18^ a minimum sample size of 18 participants was estimated. Taking into account a possible sample loss of 10% and adopting a sample with a 1:2 distribution, 20 patients for the case group and 40 for the control group were necessary.

### Evaluation procedures

Initially, anamnesis, overall physical and otoneurological examination were performed. The following were considered as otologic symptoms: self-report of presence of tinnitus (sound perception in the absence of external sound stimulus),^19^ aural fullness (full ear sensation), otalgia and hearing loss. The otoneurological examination started with the assessment of static balance, using the Romberg test, and dynamic balance, using the Fukuda test.^20^ Nystagmus (spontaneous and semi-spontaneous) was assessed, in addition to the head shaking, head impulse, Dix-Hallpike tests, and the roll test, especially when benign paroxysmal positional vertigo (BPPV) was suspected.^21^ Cerebellar function was assessed by the finger-to-nose and diadochokinesis tests.^22^

To evaluate the impact of dizziness on the volunteers’ quality of life, the Dizziness Handicap Inventory (DHI) adapted into Portuguese was used.^23^ It consists of 25 items related to physical, emotional and functional aspects. The patients answer “yes”, “sometimes” or “no” to each question, assigning four, two or zero points, respectively.^24^ The higher the score, the worse the quality of life. The DHI score analysis classifies the self-perception of dizziness as mild when the score is between zero and 30; moderate, when between 31 and 60; and severe, when between 61 and 100.^25^

As complementary exams, biochemical evaluations, pure tone audiometry, vocal audiometry, impedanciometry and the Video Head Impulse Test (VHIT) were performed. To define the type and degree of hearing loss, classifications of Silman and Silverman^26^ and the World Health Organization (WHO)^27^ were used, respectively. For the impedanciometry, the Jerger’s tympanometric classification ^28^ was used in order to evaluate the mobility of the tympanic-ossicular system. For the VHIT, the normal limits of the Vestibulo-Ocular Reflex (VOR) gain were standardized between 0.99 and 1.09 degrees per second for the lateral canals and between 0.87 and 1.21 degrees per second for the vertical canals.^29^ Moreover, an otoscopy was performed in all patients to check for impacted cerumen or other objects in the ear canal, which could require their removal to relieve symptoms of vertigo and for an adequate audiometric test.^22^

### Data analysis

As a case-control study, to ensure greater comparability between the groups, they were matched by gender, seeking a proportion of 75% of women and 25% of men in both groups, and by age, so that the means of the ages in the two groups were not significantly different. Analyses were made by the Student’s *t*-test.

Statistical analyses were performed using the SPSS 20.0 program. The Shapiro-Wilk test was performed to verify the normality of the data. The Chi-Square test and the independent *t*-test were applied for variables with normal distribution and the Mann-Whitney and Kruskal-Wallis test for non-normal variables. Therefore, values in the 95% confidence interval and p < 0.05 were considered as having statistical significance.

## Results

In the case group, which included 20 individuals, the mean age of the group was 36.3 ± 12.3 years, with 75% of female patients. The control group consisted of 40 individuals, with a mean age of 43.05 ± 13.20 years, 72.5% of which were female. The groups were homogeneous regarding the mean age (*p* =  0.06).

**Figure 1:**
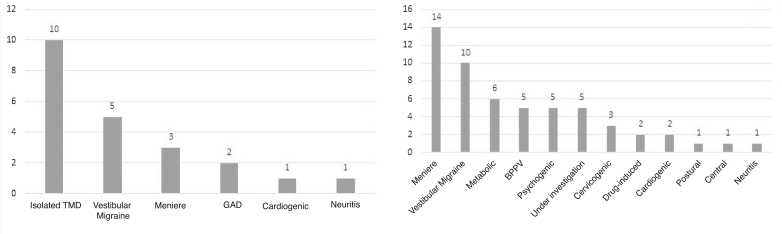
Frequency of diagnoses associated with dizziness in patients in the case (A) and control (B) groups.

Of the patients in the case group (n = 20), 50% (n = 10) had only TMD as a probable cause for dizziness (Fig. 1A), and the other patients had other associated diagnosis, such as Meniere’s disease, vestibular migraine, cardiogenic dizziness and benign paroxysmal positional vertigo (BPPV), generalized anxiety disorder (GAD) and vestibular neuritis. The control group had the diagnoses identified in Fig. 1B as the probable etiology for dizziness. The same patient may had more than one diagnosis.

**Figure 2:**
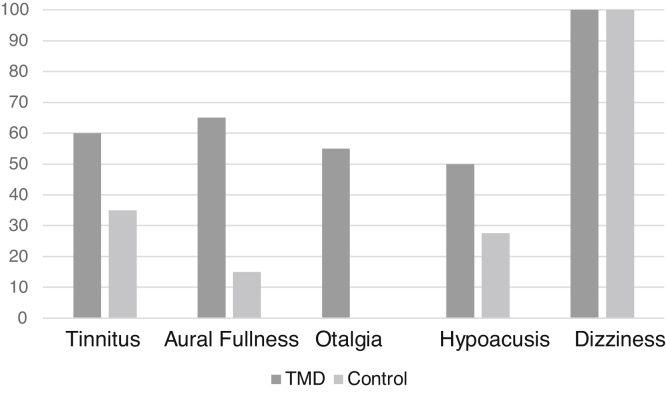
Distribution of symptoms between cases and controls.

Regarding the clinical evaluation, the most frequent symptom in the sample (excluding dizziness, which was present in all patients evaluated as it was an inclusion criterion) was tinnitus, in 26 patients (43.3%). In the case group, the most frequent symptom was aural fullness (Fig. 2).

Moreover, a statistically significant difference was found regarding aural fullness and otalgia symptoms between the groups (*p* < 0.01) in the present study (Table 1).

**Table 1:** Frequency of otoneurological symptoms between patients with and without TMD.

Symptoms	Yes	No	*p*
Tinnitus			0.06^a^
Case	12 (60%)	8 (40%)	
Control	14 (35%)	26 (65%)	
Aural fullness			<0.01^a^
Case	13 (65%)	7 (35%)	
Control	6 (15%)	34 (85%)	
Otalgia			<0.01^a^
Case	11 (55%)	9 (45%)	
Control	0 (0%)	40 (100%)	
Hypoacusis			0.08^a^
Case	10 (50%)	10 (50%)	
Control	11 (27.5%)	29 (72.5%)	

a Chi-Square Test; *p* < 0.05.

Despite the reported symptoms, no changes were found in the otoscopy of any of the individuals. On the other hand, the audiometry was normal in 90% of the patients in the case group and in 46.15% of the patients in the control group. Therefore, an association was found between TMD and a normal audiometry (*p* < 0.01).

The only altered findings in the physical examination of the patients in the case group were Fukuda (30%) and Dix Hallpike (5%) tests, which were associated with patients without pure TMD, that is, confirming that these alterations were not due to TMD, but due to associated vestibulopathies. In the control group, a greater number of alterations were observed in the otoneurological physical examination. These findings, however, showed no statistical difference when the two groups were compared.

The VHIT findings were normal in 65% of the patients in the case group and in 45% of the control group and there was no association between having TMD and vestibular alterations in the VHIT (*p* = 0.12). The VHIT assessment was based on the vestibulo-ocular reflex gain values ​​and abnormal results (hypofunctions) were considered when these gain values ​​were below the reference ones. In the case group, hypofunctions (n = 7) were more often identified in the anterior semicircular canals (5 cases), followed by the posterior canal (3 cases), and of these, there was one patient with two affected canals (one anterior and one posterior), but none had superior canal hypofunction.

**Figure 3:**
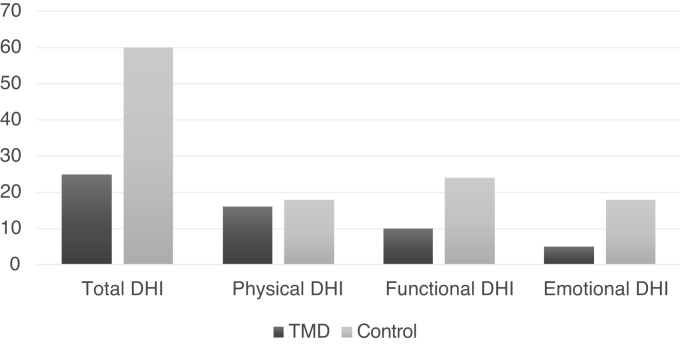
Total DHI median and subtypes in the case and control groups.

The medians of the total DHI and the respective domains are shown in Fig. 3 for both groups.

Significant differences were observed in the total DHI and in the functional and emotional domains (*p* < 0.01), being greater in the control group (Table 2).

**Table 2:** Comparison of DHI medians and IQR by domain, between groups.

	Total DHI	Physical DHI	Functional DHI	Emotional DHI
**TMD**	25 (20.5–43)	16 (8.5–19.5)	10 (6–15.5)	5 (2–10)
**Control**	60 (42–72)	18 (12–22)	24 (18–28)	18 (10–24)
***p***	<0.001^a^	=0.068^a^	<0.001^a^	<0.001^a^

a Mann-Whitney Test.

Regarding the types of TMD (articular, muscular and mixed), there was no association with total DHI (*p* = 0.892), or with the physical, functional and emotional domains.

## Discussion

Several studies have shown association between otoneurological symptoms and TMD; however, the physiopathogeny that justifies this association remains uncertain.^2^, ^12^, ^15^ Among the possible hypotheses, the anatomical proximity and embryological relationship of the ear and the masseter muscle stand out, which can generate compression of vessels, nerves and ligaments when the TMJ is not adequately positioned.^2^, ^12^, ^18^

Due to the multifactorial aspect of TMD,^30^ it is difficult to identify the main cause for these symptoms, which may be associated with middle and inner ear dysfunctions, as well as functional disorders of the mastication organ.^18^ Therefore, a multidisciplinary approach to the diagnosis and treatment of this condition is crucial.^7^ This study included an extensive evaluation made by dentists, physical therapists, speech therapists and otorhinolaryngologists, in an attempt to make the diagnosis more accurate and to reduce the selection bias.

The highter prevalence of TMD in women found in this study corroborates the findings of the literature.^18^, ^31^, ^32^ The most prevalent symptom in the two groups was tinnitus. The case group had higher frequency of aural fullness and otalgia, in agreement with the findings of Toledo et al.^7^ The most common secondary cause of otalgia is the temporomandibular disorder.^33^ The complexity in the embryonic development of the ear results in neural connections with several cranial and cervical nerves, which in turn provide sensory innervations to the head region. Trigeminal nerve pain is the most common source of secondary otalgia due to TMJ alterations.^34^

Aural fullness is a common otological complaint, but alterations are usually observed in complementary exams, such as tympanometry or audiometry. When clinical complains are reported, but no alterations in these exams are found, one should question the patient about the existence of parafunctional habits and perform a complete physical examination of the TMJ evaluation. Therefore, the evaluation of the TMJ becomes important as a differential diagnosis for other conditions, such as obstruction by cerumen, otitis or tubal dysfunction.

In our study, all patients had normal otoscopy, which may be a positive point, since we excluded cases of otitis media or external, which could be confounding factors. As for the otoneurological physical examination, the findings were similar between the groups. Half of the patients with TMD did not have a diagnosis of associated vestibulopathy, suggesting this dysfunction as a probable etiological factor for dizziness.

Moreover, we found an association between TMD and a normal audiometry, confirming that the symptom of aural fullness is not due to otological factors, but to para-auditory factors. Additionally, this finding is controversial in the literature. The study by Effat^31^ identified sensorineural losses in patients with TMD, while Totta et al.^35^ found no changes in the audiometry of these patients. Most of the studies, however, believe that patients with TMD may have associated cochlear pathologies that can cause alterations in the audiometry and/or tympanometry, so that these alteration findings could suggest cases in which pure TMD does not occur.

Dizziness is found with some frequency in patients with TMD and has an important impact on quality of life.^7^ Two possible mechanisms attempt to explain this relationship. One of them is the vascular compression of the internal auditory and posterior auricular arteries, which can occur as a result of painful stimuli to the peridiscal tissues, which would generate a sympathomimetic reflex, causing a reduction in the blood supply to the middle and inner ears.^33^ Another explanation is related to spastic contractions of the stapedius muscle, which would cause sudden and severe movements in the stapes platform, initiating waves in the perilymph of the labyrinth, producing a dizziness sensation.^36^ Moreover, the mandibular condyle can cause compression of the auriculotemporal nerve, due to its anatomical proximity, promoting a contracture of the tensor tympani muscle.^13^, ^18^

The VHIT can be used in the objective evaluation of dizziness, which is a very sensitive test used to evaluate the semicircular canals to detect peripheral vestibular dysfunctions. In our findings, there was no association between having TMD and having vestibular alterations in the VHIT. Only 35% of the patients in the case group of the present study had alterations in the VHIT, and of these, the majority had associated vestibulopathy; therefore, it can be concluded that these alterations were possibly associated with other diseases found in these patients. Taking into account the influence of the somatosensory system on the vestibular system, the study by Grande-Alonso et al.^37^ did not identify any significant vestibular dysfunction in patients with cervicogenic dizziness, which corroborates the findings of the present study.

One of the ways to assess the impact generated by dizziness on the patient’s life is through the DHI, which is subdivided into three domains: physical, emotional and functional. The functional sphere detects impaired performance regarding the professional, domestic, social and leisure aspects. The emotional sphere, on the other hand, studies the fear of leaving home or staying at home alone, shame of clinical symptoms, self-image concerns, difficulty focusing, feelings of incapacity, depression, and problems with family and social relationships.^23^ In our findings, the control group showed a greater impact on the functional and emotional domains, which can be justified by the fact that most of the sample had TMD as the only etiological diagnosis, while the control group had other etiological diagnoses, so that the other otoneurological diseases such as Meniere’s disease, vestibular migraine, metabolic dizziness and benign paroxysmal positional vertigo (BPPV) could have a more intense impact on the patient’s quality of life than TMD itself. It has been described in the literature that dizziness caused by Meniere’s Disease, vestibular migraine or even BPPV, for instance, can be disabling for the patient.^10^

Another justification for the greater impact of dizziness on quality of life as assessed by the DHI, being greater in the control group is the fact that more than half of the TMD patients had otalgia, which may lead the patient to have a more intense perception of discomfort due to pain than due to dizziness. Therefore, when asked about the influence of dizziness on their quality of life, patients with otalgia may have minimized the impairments caused by dizziness.

The limitations of the present study include the small sample size, although it is in agreement with our representativeness calculation, the non-inclusion of a TMD group without associated symptoms, and the fact that the control group was retrospectively obtained from medical records. On the other hand, this study included the diagnosis of TMD through an assessment tool considered the gold standard and applied by a trained dental surgeon, in addition to a comprehensive otoneurological assessment, emphasizing the importance of multidisciplinary care for individuals with associated dizziness and TMD.

## Conclusion

According to the data from the present study, aural fullness and otalgia symptoms are associated with TMD in patients with dizziness, just as there is an association between normal audiological tests and TMD. On the other hand, patients with dizziness and without TMD have a greater impact on their quality of life, in the functional (related to professional, domestic, social and leisure aspects) and emotional (related to negative feelings such as fear or shame due to dizziness) domains.

## Conflicts of interest

The authors declare no conflicts of interest.
